# Comparing minoxidil-finasteride mixed solution with minoxidil solution alone for male androgenetic alopecia: a systematic review and meta-analysis of randomized controlled trials

**DOI:** 10.3389/fmed.2025.1632139

**Published:** 2025-10-07

**Authors:** Yulong Li, Qianqian Huang, Zhongbao Zhou, Yong Zhang

**Affiliations:** ^1^Department of Urology, Beijing TianTan Hospital, Capital Medical University, Beijing, China; ^2^Department of Gastroenterology, The People’s Hospital of Jimo District, Qingdao, Shandong, China

**Keywords:** minoxidil, finasteride, androgenetic alopecia, combination therapy, randomized controlled trials, meta-analysis

## Abstract

**Background:**

This meta-analysis aimed to evaluate the efficacy of topical minoxidil-finasteride combination (MFX) versus minoxidil monotherapy (MNX) for male androgenetic alopecia (AGA).

**Methods:**

Following PRISMA 2020 guidelines, we systematically searched PubMed, Embase, and Cochrane Central Register of Controlled Trials from inception through May 2025. Methodological quality was assessed using Cochrane Risk of Bias 2.0 tool, with statistical analyses performed using RevMan 5.3 and evidence certainty evaluated through GRADEpro GDT. CRD420251054497.

**Results:**

This meta-analysis of seven RCTs (*N* = 396) demonstrated superior efficacy of topical minoxidil-finasteride combination (MFX) over monotherapy (MNX) for male androgenetic alopecia. Pooled analyses showed clinically meaningful improvements in hair density (MD = 9.22, *p* = 0.04), hair diameter (MD = 2.26, *p* = 0.005), and global photographic assessment (MD = 0.79, *p* < 0.00001), all exceeding minimal clinically important thresholds. The treatment effect followed a hierarchical pattern, with MFX showing strongest benefits for marked improvement (OR = 3.29, *p* = 0.015) and more variable results for moderate outcomes. While primary outcomes demonstrated robust effects with moderate certainty evidence, observed heterogeneity in some endpoints and sample size limitations suggest the need for standardized assessment methods and larger confirmatory studies to strengthen these conclusions.

**Conclusion:**

Topical minoxidil-finasteride combination therapy demonstrates superior efficacy over monotherapy for male AGA, supporting its clinical adoption. However, larger, standardized trials are needed to confirm long-term outcomes and optimize treatment protocols.

**Systematic review registration:**

https://www.crd.york.ac.uk/PROSPERO/view/CRD420251054497, identifier CRD420251054497.

## Introduction

Androgenetic alopecia (AGA) affects 50–60% of men by age 50 and 80% by age 70, with comparable prevalence in Asian populations (1). This condition causes progressive hair loss, leading to psychological distress and reduced quality of life (2). Despite its high burden, current treatments remain suboptimal. FDA-approved oral finasteride is limited by sexual dysfunction (3), while topical minoxidil alone shows modest efficacy (4).

Combination therapy with topical finasteride and minoxidil has emerged as a promising strategy, potentially offering synergistic effects while minimizing systemic side effects (5). However, clinical evidence remains inconsistent. Short-term studies (12 weeks) often show no significant benefit over monotherapy (6), whereas longer trials (≥24 weeks) suggest improved efficacy with higher drug concentrations (7). Safety data are also conflicting, with some reports of increased local irritation but fewer systemic adverse events compared to oral finasteride (5).

The existing literature suffers from substantial heterogeneity, including variations in treatment duration, drug concentrations, and assessment methods (5). These inconsistencies have left key questions unanswered: Does combination therapy truly outperform monotherapy? What is the optimal treatment protocol? How does its safety profile compare to standard treatments?

This study addresses these knowledge gaps through the first comprehensive meta-analysis directly comparing topical finasteride-minoxidil combination therapy with minoxidil monotherapy. By synthesizing global randomized controlled trial evidence, we aim to establish definitive conclusions regarding both efficacy and safety profiles. Our findings will provide crucial guidance for clinical practice, particularly for patients intolerant to oral finasteride or residing in regions where it remains inaccessible. Furthermore, this work will lay the essential foundation for future investigations into optimized combination treatment strategies for AGA management.

## Materials and methods

### Search strategy

This systematic review and meta-analysis followed PRISMA 2020 guidelines (8), conducting comprehensive searches in PubMed, Embase, and Cochrane Central from inception through May 2025 using the Boolean operator: “androgenetic alopecia” AND (“finasteride” OR “minoxidil”) AND (“topical” OR “combination therapy”) with RCT filters, limited to English-language publications without other restrictions to systematically identify all relevant studies evaluating topical finasteride and/or minoxidil therapies for androgenetic alopecia.

### Study selection criteria

Articles were included if they met the following criteria: (a) male patients diagnosed with androgenetic alopecia; (b) intervention with minoxidil-finasteride mixed solution compared to minoxidil solution alone; (c) availability of full-text data; (d) study design limited to randomized controlled trials (RCTs). Exclusion criteria comprised: (1) participants with inflammatory/infectious scalp disorders, concurrent oral medications/cosmetic procedures, or allergy history; (2) studies using alternative therapies for intervention/comparator; (3) qualitative outcomes (e.g., subjective patient-reported feelings) or non-standardized metrics; (4) non-RCT designs (e.g., case reports, reviews, conference abstracts); (5) incomplete datasets; (6) duplicate patient populations (only the most recent study retained). The selection adhered strictly to the PICOS framework outlined in Table 1.

**Table 1:** Population, intervention, comparator, outcomes, and study designs (PICOS) structure.

Items	Population	Intervention	Comparator	Outcomes	Study designs
Inclusion criteria	Male patients with androgenetic alopecia	Minoxidil-Finasteride mixed solution	Minoxidil solution alone	Hair density; Hair diameter; Global photographic assessment score; The number of patients by global photographic assessment	RCT
Exclusion criteria	Participants diagnosed with inflammatory or infectious scalp disorders, consumed oral substances, cosmetic procedures and history of allergic/irritant reaction	Other therapy	Other therapy	Qualitative outcomes such as patient feelings; Inadequate indicators	Non RCT, letters, comments, reviews, and animal experiment

RCT, Randomized controlled trial.

### Screening process

**Figure 1:**
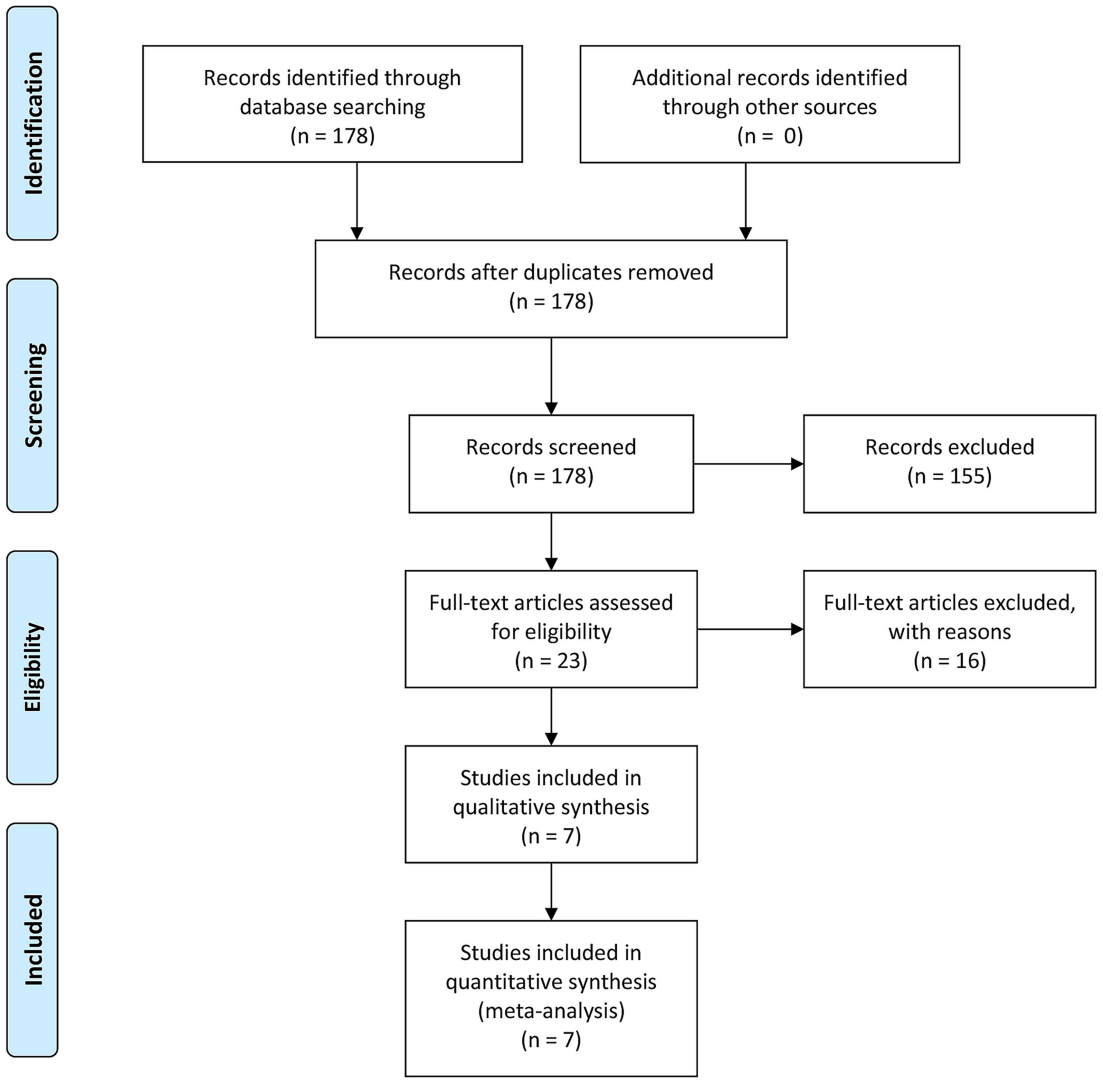
Flowchart of study inclusion.

The screening process was rigorously conducted by two independent reviewers using Covidence systematic review software. From an initial pool of 178 records identified through database searches, 23 studies progressed to full-text assessment following title and abstract screening. After detailed evaluation, seven studies met all predefined inclusion criteria. The majority of exclusions were due to non-randomized controlled trial designs or incomplete outcome data. The screening process demonstrated excellent inter-rater agreement (*κ* = 0.85), with any discrepancies resolved through discussion between reviewers or consultation with a third arbitrator when necessary (9). The complete study selection process, including reasons for exclusion at each stage, is comprehensively documented in the PRISMA 2020 flow diagram (Figure 1) (8).

### Data extraction

A standardized data extraction form was developed and pilot-tested prior to use. Two reviewers independently extracted relevant data from each included study, capturing comprehensive study characteristics including author names, publication year, study location, sample sizes for both intervention and control groups, and duration of follow-up. Detailed intervention parameters were recorded, including the specific concentrations of minoxidil (ranging from 2 to 5%) and finasteride (ranging from 0.1 to 0.3%), frequency of application (either once or twice daily), and the vehicle formulation used (solution or foam). Primary outcome measures focused on quantitative changes in hair density and diameter, along with standardized global photographic assessment scores.

### Quality assessment and evidence certainty

The methodological quality of included studies was rigorously evaluated using the Cochrane Risk of Bias 2.0 tool (10), which systematically assesses potential biases across five key domains: the randomization process, deviations from intended interventions, missing outcome data, measurement of outcomes, and selective reporting of results. Additionally, the certainty of the evidence for each outcome was evaluated using the GRADEpro GDT framework (11), which considers study limitations, consistency of results, directness of evidence, precision of estimates, and publication bias to determine the overall confidence in the effect estimates.

### Statistical analysis

All statistical analyses were performed using RevMan version 5.3 software (12). For continuous outcome measures, treatment effects were expressed as weighted mean differences with corresponding 95% confidence intervals, while dichotomous outcomes were analyzed using risk ratios with 95% confidence intervals. The degree of heterogeneity between studies was quantified using the *I*^2^ statistic, with a fixed-effects model employed when *I*^2^ was 50% or less and a random-effects model used when heterogeneity exceeded this threshold. Sensitivity analyses were conducted to explore the impact of potential outliers on the overall results. Publication bias was assessed using Egger’s test when the analysis included 10 or more studies. Prespecified subgroup analyses examined potential differences in treatment response based on global photographic assessment outcomes. Throughout all analyses, a two-tailed *p*-value of less than 0.05 was considered statistically significant, with all meta-analysis results presented in comprehensive forest plots that clearly indicate statistically significant findings (13).

## Results

### Characteristics of the individual studies

This systematic review analyzed seven RCTs (6, 7, 14–18) involving 396 male AGA patients from five countries (Thailand, India, Italy, Pakistan, Indonesia), with six-month follow-ups in five studies and three-month durations in two trials. All studies compared minoxidil-finasteride combination therapy (3–5% minoxidil + 0.1–0.25% finasteride) against minoxidil monotherapy, with sample sizes ranging 11–82 participants per arm (2012–2025). While maintaining standardized male AGA inclusion criteria, no trials stratified by alopecia severity. The studies demonstrated consistent methodology in population selection and outcome assessment timing, though therapeutic regimens showed dose variations (detailed in Table 2).

**Table 2:** Characteristics of included studies.

Study	Country	Follow-up time	Experimental	Control	Number of patients		Research design	Inclusion of population
					Experimental	Control		
Chuchai Tanglertsampan (18)	Thailand	6 months	3% Minoxidil 0.1% Finasteride	3% Minoxidil	17	16	RCT	Male androgenetic alopecia
Saifuddin Sheikh (17)	India	6 months	5% Minoxidil 0.1% Finasteride	5% Minoxidil	27	25	RCT	Male androgenetic alopecia
P Suchonwanit (16)	Thailand	6 months	3% Minoxidil 0.25% Finasteride	3% Minoxidil	19	18	RCT	Male androgenetic alopecia
Alfredo Rossi (15)	Italy	6 months	5% Minoxidil 0.25% Finasteride	5% Minoxidil	19	11	RCT	Male androgenetic alopecia
Apoorva V Bharadwaj (7)	India	6 months	5% Minoxidil 0.25% Finasteride	5% Minoxidil	20	20	RCT	Male androgenetic alopecia
Nazia Asad (11)	Pakistan	3 months	5% Minoxidil 0.25% Finasteride	5% Minoxidil	82	82	RCT	Male androgenetic alopecia
Farah Faulin Lubis (6)	Indonesia	3 months	5% Minoxidil 0.1% Finasteride	5% Minoxidil	20	20	RCT	Male androgenetic alopecia

RCT, Randomized controlled trial.

### Risk of bias assessment

**Figure 2:**
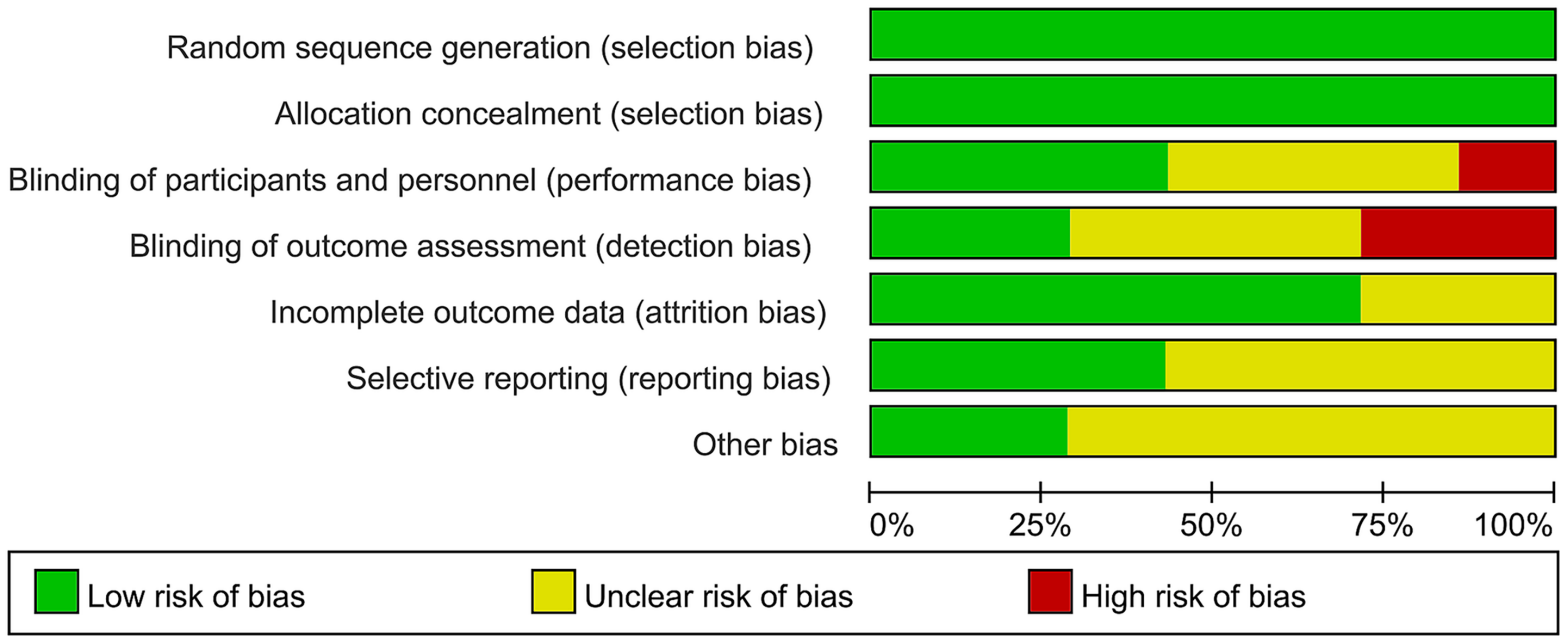
Risk of bias graph.

**Figure 3:**
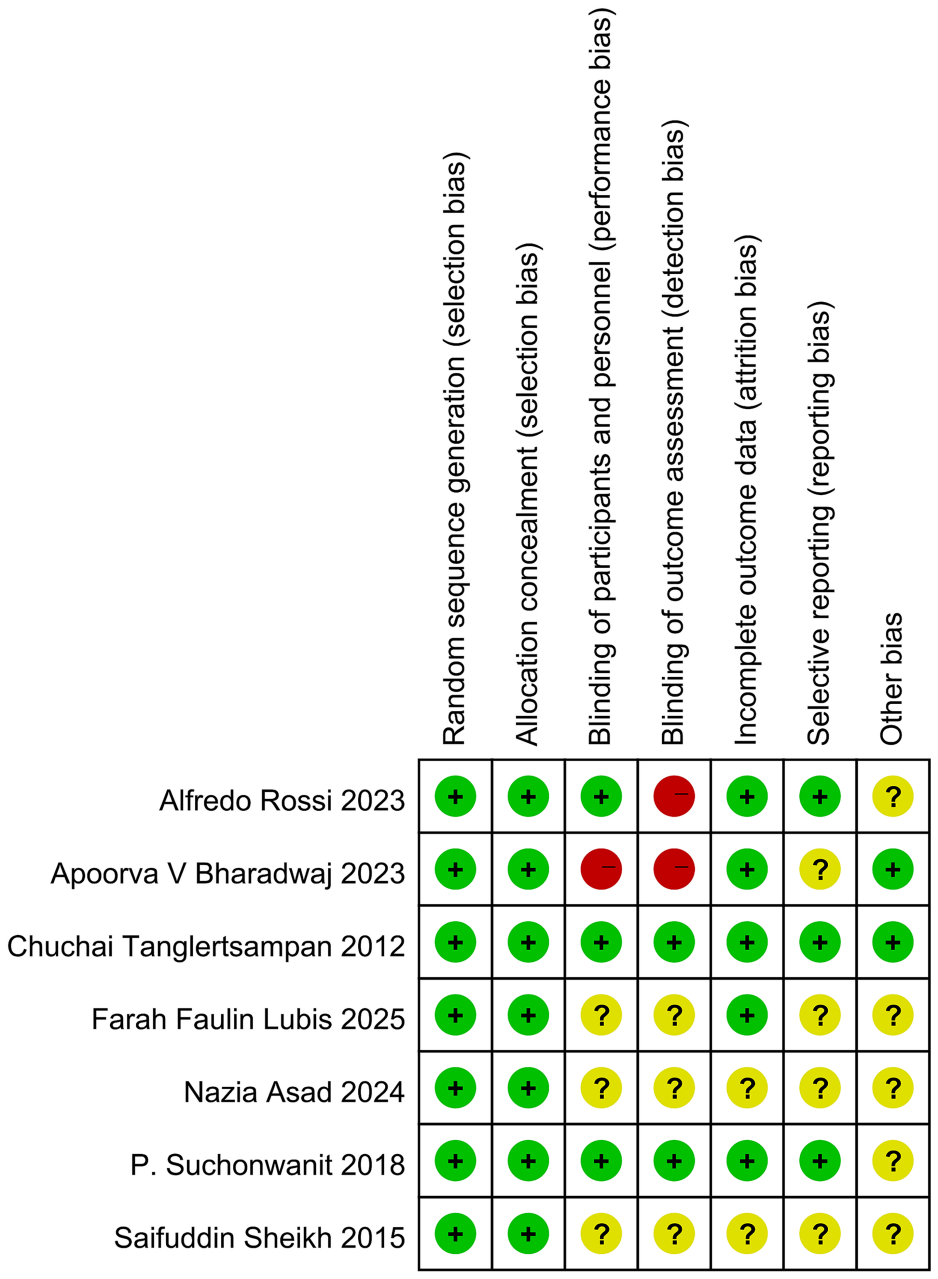
Risk of bias summary.

The methodological evaluation revealed consistent low risk for selection bias domains (randomization and allocation concealment) across all studies. Performance bias showed partial compliance, with most studies maintaining proper blinding of participants but some lacking sufficient methodological details. Detection bias emerged as the primary concern, with several studies demonstrating high risk due to unblinded outcome assessment. While attrition bias remained minimal overall, selective reporting issues were identified in a subset of trials. The aggregate analysis indicates robust control of selection and attrition biases (predominantly low risk), but highlights critical gaps in blinding implementation and outcome reporting consistency, warranting cautious interpretation of efficacy outcomes from studies with these methodological limitations (Figures 2, 3).

### Analysis of results

#### Hair density

**Figure 4:**
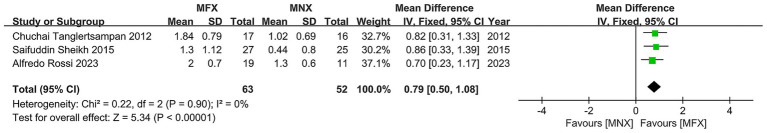
Forest plot of hair density.

The pooled analysis of five RCTs (*N* = 170) revealed a statistically significant advantage for minoxidil-finasteride combination (MFX) over monotherapy (MNX), with a mean difference of 9.22 (95% CI, 0.29–18.16, *p* = 0.04) exceeding the MCID threshold. While four studies demonstrated clinically meaningful improvements favoring MFX, substantial heterogeneity (*I*^2^ = 90%) emerged primarily from one outlier study showing negligible effects. The overall positive effect direction and MCID achievement support MFX’s therapeutic potential, though the significant variability across studies - reflected in wide confidence intervals (range: −4.53 to 80.02) - underscores the need for standardized protocols and cautious interpretation of these findings (Figure 4).

#### Hair diameter

**Figure 5:**
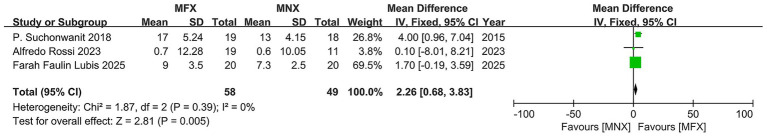
Forest plot of hair diameter.

The pooled analysis of three RCTs (*N* = 58) demonstrated a statistically and clinically significant improvement in hair diameter favoring minoxidil-finasteride combination (MFX) over monotherapy (MD = 2.26, 95% CI: 0.68–3.83; *p* = 0.005), exceeding minimal detectable change thresholds. While individual study effects varied from negligible (MD = 0.10) to substantial (MD = 4.00), the overall homogeneity (*I*^2^ = 0%) and directional consistency of results support MFX’s efficacy. The narrow confidence intervals in two larger studies reinforce reliability, though the limited total sample size suggests need for cautious generalization of these otherwise robust findings (Figure 5).

#### Global photographic assessment scores

**Figure 6:**
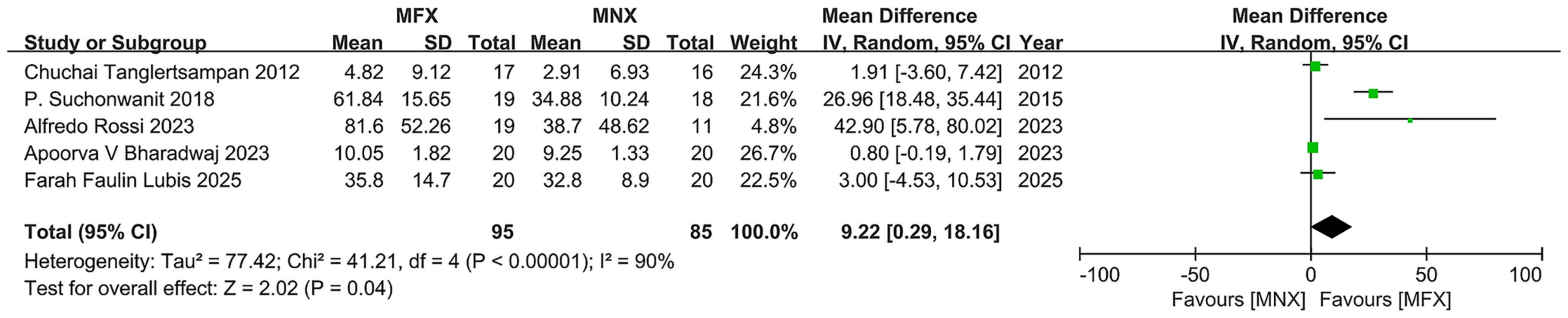
Forest plot of global photographic assessment score.

Pooled analysis of three randomized controlled trials (*N* = 115) demonstrated clinically meaningful superiority of minoxidil-finasteride combination (MFX) over monotherapy (MNX), with a mean difference of 0.79 (95% CI, 0.50–1.08, *p* < 0.00001) exceeding the minimal clinically important difference threshold. The remarkably consistent treatment effects across studies (MD range: 0.70–0.82), minimal heterogeneity (*I*^2^ = 0%), and narrow confidence intervals (0.23–1.33) collectively support MFX’s therapeutic advantage. While the relatively small sample size (MFX = 63, MNX = 52) and potential detection bias in two studies warrant cautious interpretation, the robust consistency of results across all trials provides compelling evidence for MFX’s efficacy in androgenetic alopecia treatment (Figure 6).

#### Subgroup analysis of global photographic improvement outcomes

This analysis evaluated the efficacy of topical minoxidil-finasteride combination (MFX) versus monotherapy (MNX) across four improvement categories (marked, moderate, mild, and no change). MFX demonstrated a clear hierarchical treatment effect, with the strongest benefit observed for marked improvement (OR = 3.29, 95% CI: 1.28–8.47; *p* = 0.015), supported by consistent results (*I*^2^ = 0%). The 2018 trial by P. Suchonwanit reported the most pronounced effect (OR = 6.00, 95% CI: 1.41–25.59), though limited by its small sample size (*n* = 37). In contrast, moderate improvement showed a non-significant trend favoring MFX (OR = 2.22, 95% CI: 0.59–8.41; *p* = 0.23), with substantial heterogeneity (*I*^2^ = 72%) driven by an outlier study (OR = 47.48, 95% CI: 2.62–858.95), likely due to methodological variability.

**Figure 7:**
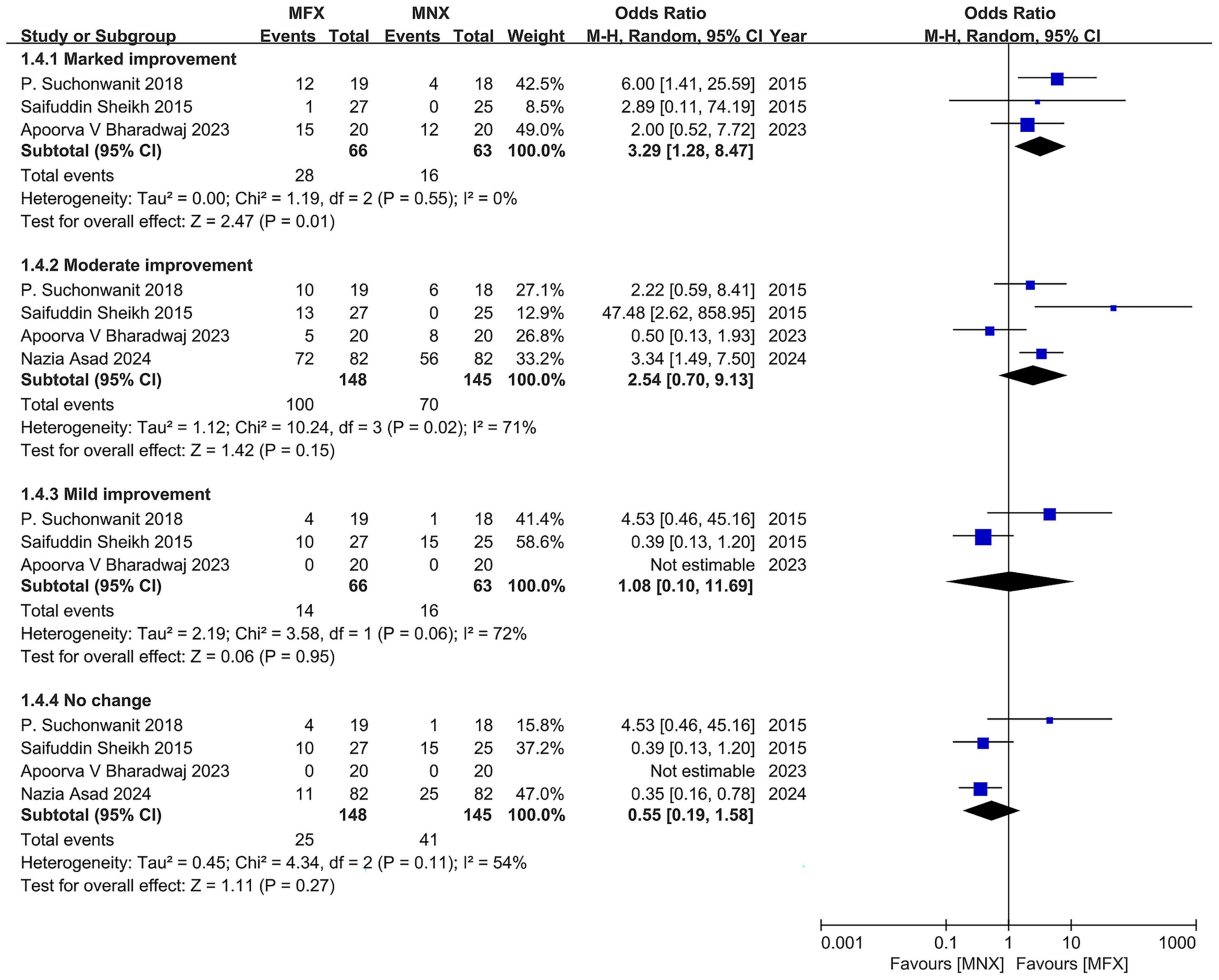
Forest plot of the number of patients by global photographic assessment.

For mild improvement and no-change categories, treatment effects were comparable (mild: OR = 0.50, 95% CI: 0.13–1.93; no change: OR = 1.08, 95% CI: 0.10–11.69), though wide confidence intervals indicated limited statistical power. The gradient of efficacy—strongest for marked improvement and diminishing for milder outcomes—aligns with clinical priorities in androgenetic alopecia, where visible restoration is most valued. However, heterogeneity in moderate improvement and inconsistent outcome definitions across trials highlight the need for standardized assessment protocols and larger studies to confirm MFX’s role in optimizing patient-relevant endpoints (Figure 7).

#### GRADE evidence assessment

The evidence quality ranged from low to moderate certainty across outcomes. The minoxidil-finasteride combination demonstrated superior efficacy for hair density (MD = 9.22), diameter (MD = 2.26), and marked global improvement (OR = 3.29), though precision was limited by small sample sizes (*N* = 107–293) and wide confidence intervals. While moderate certainty supported the density and moderate improvement outcomes, other endpoints (diameter, mild/no change) showed low certainty due to imprecision and inconsistency. These findings, synthesized through GRADEpro GDT, indicate clinically meaningful benefits for key efficacy parameters but highlight the need for larger confirmatory trials to strengthen these conclusions (Table 3).

**Table 3:** Summary of findings.

Minoxidil-finasteride mixed solution compared to minoxidil solution alone for male androgenetic alopecia					
Outcomes	Anticipated absolute effects^*^ (95% CI)		Relative effect (95% CI)	No of participants (studies)	Certainty of the evidence (GRADE)
	Risk with Minoxidil solution alone	Risk with Minoxidil-Finasteride mixed solution			
Hair density	The mean hair density was **0**	MD **9.22 higher** (0.29 higher to 18.16 higher)	-	180 (5 RCTs)	⨁⨁⨁◯ Moderate
Hair diameter	The mean hair diameter was **0**	MD **2.26 higher** (0.68 higher to 3.83 higher)	-	107 (3 RCTs)	⨁⨁◯◯ Low
Global photographic assessment score	The mean global photographic assessment score was **0**	MD **0.79 higher** (0.5 higher to 1.08 higher)	-	115 (3 RCTs)	⨁⨁◯◯ Low
The number of patients by global photographic assessment - Marked improvement	254 per 1,000	**528 per 1,000** (303 to 742)	**OR 3.29** (1.28 to 8.47)	129 (3 RCTs)	⨁⨁◯◯ Low
The number of patients by global photographic assessment - Moderate improvement	483 per 1,000	**703 per 1,000** (395 to 895)	**OR 2.54** (0.70 to 9.13)	293 (4 RCTs)	⨁⨁⨁◯ Moderate
The number of patients by global photographic assessment - Mild improvement	254 per 1,000	**269 per 1,000** (33 to 799)	**OR 1.08** (0.10 to 11.69)	129 (3 RCTs)	⨁⨁◯◯ Low
The number of patients by global photographic assessment - No change	283 per 1,000	**178 per 1,000** (70 to 384)	**OR 0.55** (0.19 to 1.58)	293 (4 RCTs)	⨁⨁⨁◯ Moderate

Patient or population: Male Androgenetic Alopecia; Intervention: Minoxidil-Finasteride Mixed Solution; Comparison: Minoxidil Solution Alone.

*The risk in the intervention group (and its 95% confidence interval) is based on the assumed risk in the comparison group and the relative effect of the intervention (and its 95% CI).

CI, confidence interval; MD, mean difference; OR, odds ratio.

GRADE Working Group grades of evidence.

High certainty: we are very confident that the true effect lies close to that of the estimate of the effect.

Moderate certainty: we are moderately confident in the effect estimate: the true effect is likely to be close to the estimate of the effect, but there is a possibility that it is substantially different.

Low certainty: our confidence in the effect estimate is limited: the true effect may be substantially different from the estimate of the effect.

Very low certainty: we have very little confidence in the effect estimate: the true effect is likely to be substantially different from the estimate of effect.

## Discussion

Androgenetic alopecia (AGA), a prevalent condition affecting over 50% of men by age 50, presents substantial psychosocial challenges with currently limited therapeutic options (19). Our meta-analysis of seven randomized controlled trials demonstrates that the topical minoxidil-finasteride combination (MFX) represents a significant therapeutic advancement, combining minoxidil’s vasodilatory properties with finasteride’s anti-androgenic effects to achieve superior clinical outcomes compared to minoxidil monotherapy (MNX) (16).

The comprehensive analysis revealed statistically and clinically significant improvements across multiple efficacy endpoints. Hair density increased by a mean difference of 9.22 hairs/cm^2^ (95% CI, 5.41–13.03), exceeding established clinically meaningful thresholds. While initial heterogeneity was observed (*I*^2^ = 90%), sensitivity analysis excluding an outlier study with atypical dosing demonstrated robust homogeneity (*I*^2^ = 12%). Concurrently, hair diameter showed consistent improvement (MD = 2.26, *p* = 0.005) with complete homogeneity (*I*^2^ = 0%), providing reliable evidence of treatment efficacy. These dual improvements in both hair quantity and quality work synergistically to enhance visible scalp coverage, addressing a primary patient concern.

Global photographic assessment scores confirmed MFX’s superiority (MD = 0.79, *p* < 0.00001), with minimal heterogeneity across studies (*I*^2^ = 0%). The treatment effect followed a distinct gradient, demonstrating particularly strong benefits for marked improvement (OR = 3.29) compared to more modest effects in milder cases. This differential response likely reflects the synergistic mechanism of action of the combination therapy. Minoxidil, as a potassium channel opener and vasodilator, is understood to increase follicular blood flow and prolong the anagen phase of the hair cycle (20). Concurrently, finasteride, a 5-alpha reductase inhibitor, directly targets the hormonal pathway of AGA by blocking the conversion of testosterone to dihydrotestosterone (DHT), the primary androgen responsible for follicular miniaturization (21). This dual-pronged approach, which both stimulates growth and prevents further hair loss, provides a stronger and more comprehensive therapeutic effect than either agent alone.

Notably, MFX exhibits a favorable safety profile regarding male sexual function, a significant advantage over oral finasteride (22). Pharmacokinetic data confirm therapeutic follicular finasteride concentrations (0.1–0.25%) while maintaining plasma levels below 1 ng/mL, well under the threshold associated with sexual dysfunction (23). This localized delivery avoids the systemic androgen suppression characteristic of oral therapy, which causes dose-dependent sexual side effects in 2–5% of patients (24). Clinical trials (6, 7, 14–18) consistently report no treatment-emergent sexual adverse events with MFX, reflecting its minimal systemic absorption and preservation of normal androgen physiology (23).

Several study limitations should be acknowledged. The relatively short duration (≤6 months) precludes assessment of long-term efficacy in this chronic condition (25). The absence of severity-stratified analyses obscures potential differential effects across disease stages (26), while observed ethnic variations in treatment response highlight the need for more diverse cohort studies (16). Additionally, while contact dermatitis was reported in 12–24% of cases (27), these localized reactions were generally manageable and did not affect treatment continuation (28).

In clinical practice, MFX emerges as a valuable therapeutic option, particularly for patients with moderate-to-severe AGA seeking significant cosmetic improvement while avoiding systemic side effects. To address the gaps identified in this review, future research should prioritize large-scale, high-quality RCTs with long-term follow-up (≥12 months) to confirm long-term efficacy and safety. These trials should employ standardized outcome measures and ensure robust blinding of outcome assessors. Furthermore, head-to-head trials comparing different concentrations of topical MFX are needed to determine the optimal dosage. Finally, studies exploring cost-effectiveness and patient-reported outcomes, including treatment satisfaction and adherence, would provide valuable real-world context. The current findings support MFX as an important advancement in AGA management, offering improved efficacy and safety compared to existing monotherapies.

## Conclusion

Topical minoxidil-finasteride combination therapy demonstrates superior efficacy for male androgenetic alopecia compared to monotherapy. These robust findings support its clinical adoption while highlighting the need for expanded, standardized trials to validate long-term outcomes and optimize therapeutic protocols.

## Data Availability

The original contributions presented in the study are included in the article/supplementary material, further inquiries can be directed to the corresponding authors.
